# (Aceto­nitrile-κ*N*)aqua­[*N*,*N*′-bis­(pyridin-2-yl­methyl)ethane-1,2-di­amine-κ^4^ *N*,*N*′,*N*′′,*N*′′′]zinc(II) perchlorate

**DOI:** 10.1107/S2056989017013603

**Published:** 2017-09-29

**Authors:** Ugochukwu Okeke, Yilma Gultneh, Ray J. Butcher

**Affiliations:** a Department of Chemistry, Howard University, 525 College Street NW, Washington, DC 20059, USA

**Keywords:** crystal structure, zinc(II), coordination complex, ethane-1,2-di­amine

## Abstract

The structure of the title compound, [Zn(C_14_H_18_N_4_)(C_2_H_3_N)(H_2_O)](ClO_4_)_2_, contains a six-coordinate cation consisting of the tetra­dentate bis­picen ligand, coordinated water, and coordinated aceto­nitrile, with the latter two ligands adopting a *cis* configuration. There are two formula units in the asymmetric unit. Both cations show almost identical structural features with the bis­picen ligand adopting the more common *cis*-β conformation. One of the four perchlorate anions is disordered over two positions, with occupancies of 0.9090 (15) and 0.0910 (15). There is extensive inter-ionic hydrogen bonding between the perchlorate anions and O—H and N—H groups in the cations, including a bifurcated hydrogen bond between an N—H group and two O atoms of one perchlorate anion. As a result of this extended hydrogen-bond network, the ions are linked into a complex three-dimensional array.

## Abstract 2

The structure of aceto­nitrile­aqua­[1,2-bis­(pyridin-2-ylmeth­yl)ethane-1,2-di­amine]­zinc(II) perchlorate contains a six-coordinate cation consisting of the tetra­dentate bis­picen ligand, coordinated water, and coordinated aceto­nitrile with the latter two ligands adopting a *cis* configuration.

## Chemical context   

One of the greatest challenges in synthetic chemistry is the selective conversion of non-activated C—H bonds to useful functional groups (Gunay & Theopold, 2010 ▸). Coordination complexes have been extensively explored due to their potential to catalyze such transformations. The ligand’s chelation around the metal ion determines the number and relative orientation of vacant coordination sites where terminal oxidants and/or substrates can bind. Installed steric bulk or substrate binding groups can either preclude certain mol­ecules from accessing the active site (Chen & White, 2010 ▸) or attract compounds with specific shapes or functional groups (Das *et al.*, 2006 ▸). These benefits rely upon the ability to understand, predict, and control the coordination geometry of the polydentate ligand.
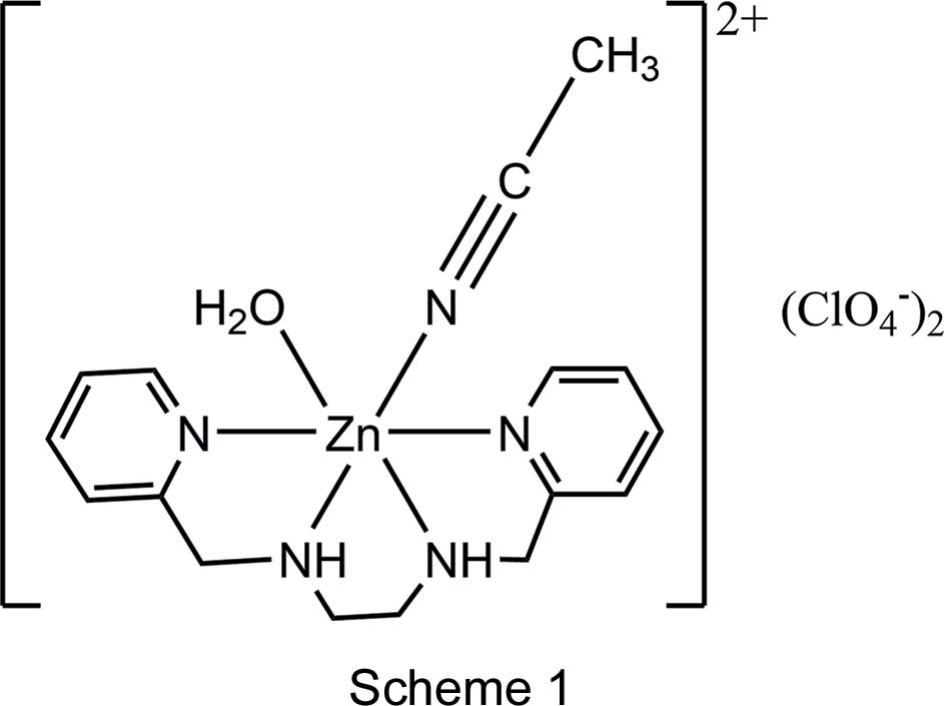


The 1,2-bis­(pyridin-2-ylmeth­yl)ethane-1,2-di­amine (bis­picen) ligand and other tetra­dentate ligands with reduced imine linkages have been observed to wrap around single transition metal ions in primarily two fashions: *cis*-α and *cis*-β (Scheme 2) (Chen *et al.*, 2002 ▸). The *cis*-α, in which the two pyridine groups are trans to each other, has been the only conformation heretofore observed with bis­picen and its methyl­ated derivatives (Goodson *et al.*, 1990 ▸, 1991 ▸). The *cis*-β conformation, in which the two pyridine groups are *cis* to each other, has been observed most often with ligands with propane-1,3-di­amine backbones (England *et al.*, 2007 ▸; Hureau *et al.*, 2005*a* ▸,*b* ▸).

A third conformational possibility, alternately described as *trans* or *planar* (Scheme 2), has been structurally observed most commonly for tetra­dentate ligands with imine linkages, such as salens (Jacobsen *et al.*, 1991 ▸). In tetra­dentate ligands with reduced imine linkages, the *trans* conformation has been observed rarely and only with ligands with either severely strained bridges or longer alkyl linkages between the amines (Mas-Ballesté *et al.*, 2006 ▸). Consequently, the *trans* conformer is rarely mentioned as a plausible isomer in reactivity studies involving bis­picen derivatives.
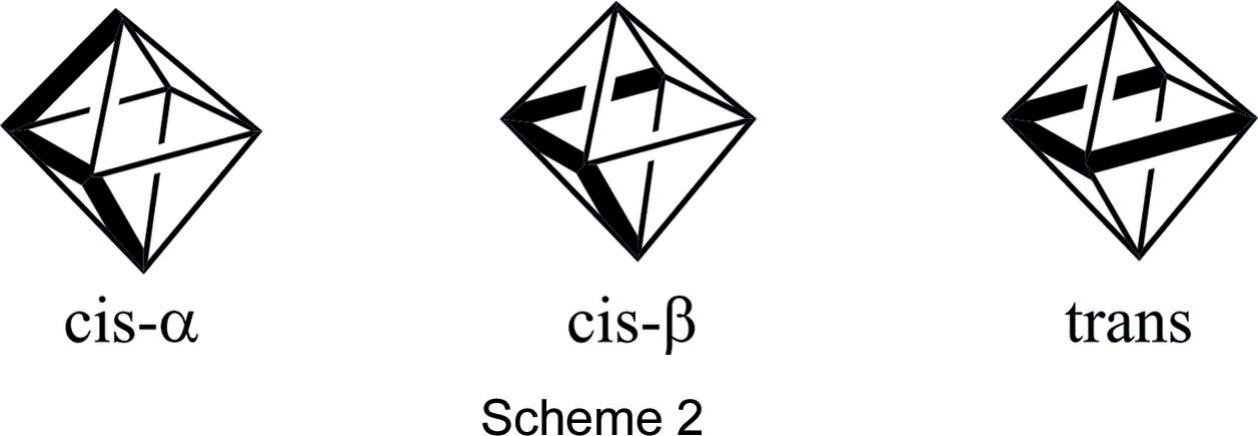


In view of the fact that Zn^II^ is a *d* ^10^ system and thus has a relatively plastic coordination environment it is of inter­est to determine which of the possible conformations the bis­picen ligand adopts upon coordination to this metal ion. There is only one previous structural study of a bis­picen derivative of Zn (Parajón-Costa *et al.*, 2013 ▸). This study is in continuation of our past studies on the role of zinc in hydrolytic enzymes (Gultneh *et al.*, 1996 ▸, 1999 ▸), in particular the role of Zn in lowering the p*K*_a_ of coordinated water mol­ecules.

## Structural commentary   

**Figure 1:**
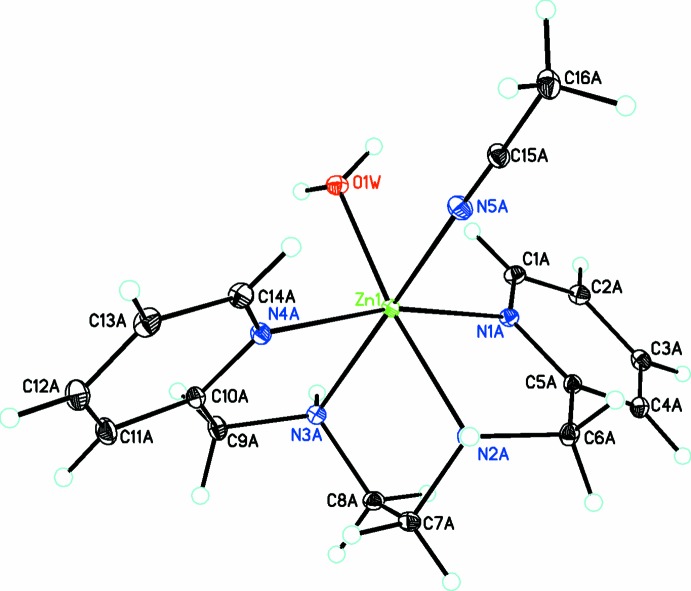
Diagram of the Zn-containing cation, showing the atom labeling. Anions have been omitted for clarity. Atomic displacement parameters are at the 30% probability level.

In the structure of the title compound (Fig. 1 ▸), the six-coordinate cation consists of the tetra­dentate bis­picen ligand, coordinated water, and coordinated aceto­nitrile, with the latter two ligands adopting a *cis* conformation. There are two complete formula units in the asymmetric unit. Both cations show almost identical structural features with the bis­picen ligand adopting the more common *cis*-β conformation, in which the two pyridine groups are *cis* to each other with the H_2_O and CH_3_CN ligands in *trans* position to the N—H groups. One of the four perchlorate anions is disordered over two positions with occupancies of 0.9090 (15) and 0.0910 (15). A related complex containing a [*cis*-Zn(bis­picen)Cl(H_2_O)]^+^ cation and a [ZnCl_4_]^2−^ anion has been published recently (Parajón-Costa *et al.*, 2013 ▸). In contrast to previous studies, bond lengths for the two types of Zn—N bonds (Zn—N_py_ and Zn—N_N—H_) show very similar values, with the exception of those which are *trans* to the coordinated aceto­nitrile (Table 1 ▸). The latter are significantly longer [2.2056 (7) and 2.2066 (7) Å]. Inter­estingly, Zn—OH_2_ bond lengths are relatively short [2.1333 (7) and 2.1279 (7) Å] reflecting a strong Zn—O bond. Further studies will be made to see the effect of this on the p*K*_a_ of the coordinated water. There is extensive inter-ionic hydrogen bonding (Table 2 ▸) between the perchlorate anions and O—H and N—H groups in the cations including a bifurcated hydrogen bond between an N—H group and two O atoms of one perchlorate anion. As a result of this extended hydrogen-bond network the ions are linked into a complex three-dimensional array.

## Supra­molecular features   

**Figure 2:**
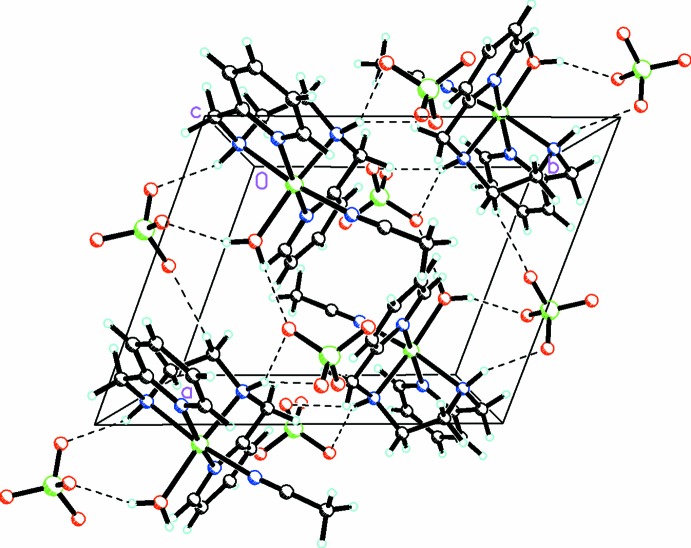
Packing diagram, viewed along the *c* axis, showing the extensive O—H⋯O, N—H⋯O, and C—H⋯O inter­actions linking the cations and anions into a complex three-dimensional array. For the disordered moieties, only the major conformation is shown.

There is a complex array of hydrogen bonds between the O—H and N—H groups in the cations and the O atoms of the anions. In addition, there are weak C—H⋯O inter­actions between the CH_3_ groups of the coordinated aceto­nitrile moieties and the adjoining O atoms of the perchlorate anions. These link the ions into a complex three-dimensional array (Fig. 2 ▸).

## Database survey   

A survey of the Cambridge Structural Database (Groom *et al.*, 2016 ▸) for complexes of bis­picen with Zn gave only one hit (Parajón-Costa *et al.*, 2013 ▸). This structure contained a [*cis*-Zn(bis­picen)Cl(H_2_O)]^+^ cation and a [ZnCl_4_]^2−^ anion.

## Synthesis and crystallization   

Pyridine-2-carbaldehyde (2.3996 g, 0.0022 mol) was added to a reaction flask and dissolved in 50 ml methanol. Ethyl­enedi­amine (0.6732 g, 0.0012 mol) was added to the solution. The mixture was stirred for 3 d before refluxing the reaction for 1 h. The contents of the reaction flask were cooled to 268 K. 4 equivalents of NaBH_4_ (1.6646 g, 0.0044 mol) were added to the reaction mixture which was then allowed to reach room temperature. The mixture was stirred overnight. Methanol was evaporated under reduced pressure. The contents were redissolved in water (50 ml) before being extracted with chloro­form (4 × 50 ml). Moisture was removed using anhydrous MgSO_4_. Chloro­form was evaporated under reduced pressure producing the ligand as a brown oil (yield 68%). The zinc(II) complex was prepared by reacting 0.4356 g (0.0018 mol) of the ligand in 50 ml aceto­nitrile with Zn(ClO_4_)_2_·6H_2_O (0.4251 g, 0.0018 mol). The mixture was stirred at room temperature overnight and layered with 50 ml diethyl ether. The container was sealed and di­ethyl­ether allowed to diffuse into the solution for 3 d to give yellow crystals which were filtered and dried [yield based on Zn(ClO_4_)_2_: 55%].

## Refinement   

Crystal data, data collection and structure refinement details are summarized in Table 3 ▸. H atoms were positioned geometrically and allowed to ride on their parent atoms, with C—H ranging from 0.95 to 0.99 Å and N—H at 1.00 Å. Displacement parameters were fixed to *U*_iso_(H) = *xU*_eq_(C), where *x* = 1.5 for methyl H atoms and 1.2 for all other C-bound and N-bound H atoms. H atoms attached to water were refined isotropically. One of the four perchlorate anions is disordered over two positions with occupancies of 0.9090 (15) and 0.0910 (15), and were constrained to have similar displacement and metrical parameters.

## Supplementary Material

Crystal structure: contains datablock(s) I. DOI: 10.1107/S2056989017013603/im2479sup1.cif

Structure factors: contains datablock(s) I. DOI: 10.1107/S2056989017013603/im2479Isup2.hkl

CCDC reference: 1576091

Additional supporting information:  crystallographic information; 3D view; checkCIF report

## Figures and Tables

**Table 1:** Selected geometric parameters (Å, °)

Zn1—N5*A*	2.1231 (8)	Zn2—O2*W*	2.1279 (7)
Zn1—O1*W*	2.1333 (7)	Zn2—N5*B*	2.1328 (8)
Zn1—N4*A*	2.1341 (7)	Zn2—N4*B*	2.1345 (7)
Zn1—N2*A*	2.1692 (7)	Zn2—N1*B*	2.1440 (7)
Zn1—N1*A*	2.1707 (7)	Zn2—N2*B*	2.1674 (8)
Zn1—N3*A*	2.2056 (7)	Zn2—N3*B*	2.2066 (7)
			
N5*A*—Zn1—O1*W*	86.48 (3)	O2*W*—Zn2—N5*B*	86.23 (3)
N5*A*—Zn1—N4*A*	93.75 (3)	O2*W*—Zn2—N4*B*	95.01 (3)
O1*W*—Zn1—N4*A*	95.55 (3)	N5*B*—Zn2—N4*B*	94.02 (3)
N5*A*—Zn1—N2*A*	92.19 (3)	O2*W*—Zn2—N1*B*	94.46 (3)
O1*W*—Zn1—N2*A*	172.06 (3)	N5*B*—Zn2—N1*B*	95.88 (3)
N4*A*—Zn1—N2*A*	92.34 (3)	N4*B*—Zn2—N1*B*	166.72 (3)
N5*A*—Zn1—N1*A*	96.07 (3)	O2*W*—Zn2—N2*B*	173.16 (3)
O1*W*—Zn1—N1*A*	93.28 (3)	N5*B*—Zn2—N2*B*	91.53 (3)
N4*A*—Zn1—N1*A*	167.17 (3)	N4*B*—Zn2—N2*B*	91.59 (3)
N2*A*—Zn1—N1*A*	79.06 (3)	N1*B*—Zn2—N2*B*	79.32 (3)
N5*A*—Zn1—N3*A*	171.11 (3)	O2*W*—Zn2—N3*B*	99.39 (3)
O1*W*—Zn1—N3*A*	99.00 (3)	N5*B*—Zn2—N3*B*	171.13 (3)
N4*A*—Zn1—N3*A*	78.83 (3)	N4*B*—Zn2—N3*B*	78.74 (3)
N2*A*—Zn1—N3*A*	83.32 (3)	N1*B*—Zn2—N3*B*	90.55 (3)
N1*A*—Zn1—N3*A*	90.62 (3)	N2*B*—Zn2—N3*B*	83.64 (3)

**Table 2:** Hydrogen-bond geometry (Å, °)

*D*—H⋯*A*	*D*—H	H⋯*A*	*D*⋯*A*	*D*—H⋯*A*
O1*W*—H1*W*2⋯O41^i^	0.80 (1)	2.04 (1)	2.8410 (12)	178 (2)
O1*W*—H1*W*2⋯O43*A* ^i^	0.80 (1)	1.94 (2)	2.730 (15)	170 (2)
N2*A*—H2*AB*⋯O41	1.00	2.28	3.1809 (12)	150
N2*A*—H2*AB*⋯O42	1.00	2.28	3.1468 (15)	144
N2*A*—H2*AB*⋯O42*A*	1.00	2.40	3.328 (12)	153
N2*A*—H2*AB*⋯O43*A*	1.00	2.50	3.396 (15)	149
N3*A*—H3*AB*⋯O23	1.00	2.17	3.0889 (12)	152
C4*A*—H4*AA*⋯O42^ii^	0.95	2.60	3.4025 (15)	142
C7*A*—H7*AA*⋯O24^iii^	0.99	2.41	3.1218 (12)	128
C14*A*—H14*A*⋯O32^iv^	0.95	2.41	3.1645 (13)	136
C16*A*—H16*B*⋯O22^v^	0.98	2.51	3.4788 (13)	169
O2*W*—H2*W*1⋯O31^iv^	0.82 (1)	2.00 (1)	2.8091 (11)	174 (2)
O2*W*—H2*W*2⋯O11	0.82 (1)	1.99 (1)	2.7690 (11)	159 (2)
N2*B*—H2*BB*⋯O31^vi^	1.00	2.36	3.2467 (12)	148
N2*B*—H2*BB*⋯O33^vi^	1.00	2.32	3.2331 (15)	152
N3*B*—H3*BB*⋯O14	1.00	2.20	3.1169 (13)	152
C7*B*—H7*BB*⋯O13^i^	0.99	2.44	3.1430 (12)	128
C14*B*—H14*B*⋯O43^i^	0.95	2.48	3.2138 (15)	134
C14*B*—H14*B*⋯O44*A* ^i^	0.95	2.32	3.149 (13)	145
C16*B*—H16*D*⋯O43*A* ^i^	0.98	2.54	3.419 (13)	150
C16*B*—H16*E*⋯O11^vii^	0.98	2.55	3.5083 (14)	166

Symmetry codes: (i) 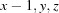; (ii) 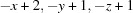; (iii) 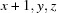; (iv) 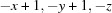; (v) 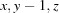; (vi) 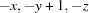; (vii) 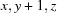.

**Table 3:** Experimental details

Crystal data	
Chemical formula	[Zn(C_14_H_18_N_4_)(C_2_H_3_N)(H_2_O)](ClO_4_)_2_
*M*_r_	565.66
Crystal system, space group	Triclinic, *P* 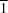
Temperature (K)	100
*a*, *b*, *c* (Å)	8.2959 (3), 10.3169 (4), 27.7884 (9)
α, β, γ (°)	92.969 (1), 98.241 (1), 109.620 (1)
*V* (Å^3^)	2204.36 (14)
*Z*	4
Radiation type	Mo *K*α
μ (mm^−1^)	1.42
Crystal size (mm)	0.45 × 0.41 × 0.29

Data collection	
Diffractometer	Bruker Quest CCD
Absorption correction	Multi-scan (*SADABS*; Sheldrick, 1996 ▸)
*T*_min_, *T*_max_	0.453, 0.753
No. of measured, independent and observed [*I* > 2σ(*I*)] reflections	37729, 37729, 31022
*R*_int_	0.047
(sin θ/λ)_max_ (Å^−1^)	1.066

Refinement	
*R*[*F* ^2^ > 2σ(*F* ^2^)], *wR*(*F* ^2^), *S*	0.042, 0.107, 1.05
No. of reflections	37729
No. of parameters	636
No. of restraints	56
H-atom treatment	H atoms treated by a mixture of independent and constrained refinement
Δρ_max_, Δρ_min_ (e Å^−3^)	2.24, −1.15

Computer programs: *APEX2* (Bruker, 2005 ▸), *SAINT* (Bruker, 2002 ▸), *SHELXT* (Sheldrick, 2015*a* ▸), *SHELXL2014* (Sheldrick, 2015*b* ▸) and *SHELXTL* (Sheldrick, 2008 ▸).

## supplementary crystallographic information

### Crystal data

**Table:**

[Zn(C_14_H_18_N_4_)(C_2_H_3_N)(H_2_O)](ClO_4_)_2_	*Z* = 4
*M**_r_* = 565.66	*F*(000) = 1160
Triclinic, *P*1	*D*_x_ = 1.704 Mg m^−^^3^
*a* = 8.2959 (3) Å	Mo *K*α radiation, λ = 0.71073 Å
*b* = 10.3169 (4) Å	Cell parameters from 9442 reflections
*c* = 27.7884 (9) Å	θ = 3.2–61.9°
α = 92.969 (1)°	µ = 1.42 mm^−^^1^
β = 98.241 (1)°	*T* = 100 K
γ = 109.620 (1)°	Block, colourless
*V* = 2204.36 (14) Å^3^	0.45 × 0.41 × 0.29 mm

### Data collection

**Table:**

Bruker Quest CCD diffractometer	31022 reflections with *I* > 2σ(*I*)
ω scans	*R*_int_ = 0.047
Absorption correction: multi-scan (SADABS; Sheldrick, 1996)	θ_max_ = 49.2°, θ_min_ = 2.3°
*T*_min_ = 0.453, *T*_max_ = 0.753	*h* = −9→9
37729 measured reflections	*k* = −12→12
37729 independent reflections	*l* = −32→33

### Refinement

**Table:**

Refinement on *F*^2^	56 restraints
Least-squares matrix: full	Hydrogen site location: mixed
*R*[*F*^2^ > 2σ(*F*^2^)] = 0.042	H atoms treated by a mixture of independent and constrained refinement
*wR*(*F*^2^) = 0.107	*w* = 1/[σ^2^(*F*_o_^2^) + (0.0391*P*)^2^ + 1.0144*P*] where *P* = (*F*_o_^2^ + 2*F*_c_^2^)/3
*S* = 1.05	(Δ/σ)_max_ = 0.006
37729 reflections	Δρ_max_ = 2.24 e Å^−^^3^
636 parameters	Δρ_min_ = −1.15 e Å^−^^3^

### Special details

**Table:**

Geometry. All esds (except the esd in the dihedral angle between two l.s. planes) are estimated using the full covariance matrix. The cell esds are taken into account individually in the estimation of esds in distances, angles and torsion angles; correlations between esds in cell parameters are only used when they are defined by crystal symmetry. An approximate (isotropic) treatment of cell esds is used for estimating esds involving l.s. planes.

### Fractional atomic coordinates and isotropic or equivalent isotropic displacement parameters (Å^2^)

**Table:**

	*x*	*y*	*z*	*U*_iso_*/*U*_eq_	Occ. (<1)
Zn1	0.83273 (2)	0.76731 (2)	0.37527 (2)	0.01022 (2)	
O1W	0.61524 (9)	0.81617 (8)	0.34185 (2)	0.01495 (10)	
H1W1	0.611 (2)	0.8958 (14)	0.3465 (7)	0.021 (4)*	
H1W2	0.5198 (19)	0.7649 (16)	0.3439 (7)	0.022 (4)*	
N1A	0.71985 (8)	0.70563 (8)	0.43995 (2)	0.01171 (9)	
N2A	1.03060 (9)	0.70258 (8)	0.41604 (3)	0.01261 (10)	
H2AB	1.0721	0.6483	0.3929	0.015*	
N3A	0.99210 (9)	0.97233 (8)	0.41415 (2)	0.01183 (9)	
H3AB	0.9157	1.0157	0.4283	0.014*	
N4A	0.99441 (9)	0.85294 (8)	0.32333 (2)	0.01277 (10)	
N5A	0.71429 (11)	0.57128 (9)	0.33332 (3)	0.01715 (12)	
C1A	0.58572 (10)	0.73342 (10)	0.45424 (3)	0.01395 (12)	
H1AA	0.5210	0.7731	0.4325	0.017*	
C2A	0.53812 (11)	0.70639 (10)	0.49963 (3)	0.01520 (13)	
H2AA	0.4410	0.7253	0.5084	0.018*	
C3A	0.63487 (11)	0.65113 (10)	0.53211 (3)	0.01514 (13)	
H3AA	0.6058	0.6325	0.5635	0.018*	
C4A	0.77454 (11)	0.62379 (10)	0.51771 (3)	0.01419 (12)	
H4AA	0.8440	0.5874	0.5393	0.017*	
C5A	0.81176 (10)	0.65040 (9)	0.47104 (3)	0.01169 (10)	
C6A	0.95559 (11)	0.61389 (10)	0.45279 (3)	0.01427 (12)	
H6AA	1.0483	0.6229	0.4808	0.017*	
H6AB	0.9094	0.5161	0.4383	0.017*	
C7A	1.17523 (10)	0.83110 (10)	0.43644 (3)	0.01500 (12)	
H7AA	1.2467	0.8654	0.4110	0.018*	
H7AB	1.2500	0.8113	0.4640	0.018*	
C8A	1.10825 (11)	0.94212 (10)	0.45419 (3)	0.01437 (12)	
H8AA	1.0441	0.9106	0.4813	0.017*	
H8AB	1.2077	1.0277	0.4669	0.017*	
C9A	1.08674 (12)	1.06278 (9)	0.38040 (3)	0.01483 (12)	
H9AA	1.2040	1.1197	0.3980	0.018*	
H9AB	1.0248	1.1264	0.3701	0.018*	
C10A	1.10454 (10)	0.98339 (9)	0.33561 (3)	0.01292 (11)	
C11A	1.22877 (12)	1.04752 (11)	0.30741 (3)	0.01845 (15)	
H11A	1.3072	1.1392	0.3173	0.022*	
C12A	1.23620 (13)	0.97543 (13)	0.26464 (4)	0.02126 (17)	
H12A	1.3189	1.0175	0.2446	0.026*	
C13A	1.12063 (13)	0.84058 (12)	0.25154 (3)	0.01897 (15)	
H13A	1.1220	0.7894	0.2223	0.023*	
C14A	1.00344 (12)	0.78261 (10)	0.28217 (3)	0.01595 (13)	
H14A	0.9266	0.6897	0.2738	0.019*	
C15A	0.66602 (11)	0.46620 (9)	0.31015 (3)	0.01465 (12)	
C16A	0.60567 (15)	0.33475 (10)	0.27988 (3)	0.01905 (15)	
H16A	0.4855	0.3148	0.2638	0.029*	
H16B	0.6110	0.2612	0.3003	0.029*	
H16C	0.6797	0.3394	0.2551	0.029*	
Zn2	0.17061 (2)	0.21312 (2)	0.12948 (2)	0.01100 (2)	
O2W	0.38318 (9)	0.15838 (8)	0.16247 (2)	0.01486 (10)	
H2W1	0.479 (2)	0.2123 (17)	0.1601 (7)	0.030 (5)*	
H2W2	0.386 (2)	0.0810 (14)	0.1559 (6)	0.019 (4)*	
N1B	0.28232 (9)	0.27927 (8)	0.06607 (2)	0.01196 (10)	
N2B	−0.02412 (9)	0.28556 (9)	0.09126 (3)	0.01434 (11)	
H2BB	−0.0633	0.3385	0.1155	0.017*	
N3B	0.00700 (9)	0.01205 (8)	0.08839 (3)	0.01278 (10)	
H3BB	0.0815	−0.0307	0.0730	0.015*	
N4B	0.00625 (9)	0.12413 (8)	0.18045 (3)	0.01389 (11)	
N5B	0.29347 (11)	0.40678 (9)	0.17350 (3)	0.01795 (13)	
C1B	0.41515 (10)	0.25042 (10)	0.05142 (3)	0.01366 (12)	
H1BA	0.4762	0.2062	0.0724	0.016*	
C2B	0.46654 (11)	0.28262 (10)	0.00690 (3)	0.01498 (13)	
H2BA	0.5621	0.2622	−0.0023	0.018*	
C3B	0.37530 (12)	0.34545 (10)	−0.02411 (3)	0.01559 (13)	
H3BA	0.4072	0.3682	−0.0549	0.019*	
C4B	0.23719 (12)	0.37432 (10)	−0.00934 (3)	0.01569 (13)	
H4BA	0.1719	0.4159	−0.0301	0.019*	
C5B	0.19527 (10)	0.34154 (9)	0.03642 (3)	0.01292 (11)	
C6B	0.05258 (12)	0.37796 (10)	0.05555 (4)	0.01661 (13)	
H6BA	0.1004	0.4746	0.0712	0.020*	
H6BB	−0.0396	0.3723	0.0278	0.020*	
C7B	−0.17097 (11)	0.15995 (11)	0.06975 (4)	0.01697 (14)	
H7BA	−0.2450	0.1837	0.0430	0.020*	
H7BB	−0.2424	0.1238	0.0950	0.020*	
C8B	−0.10838 (11)	0.04847 (10)	0.04981 (3)	0.01612 (13)	
H8BA	−0.2099	−0.0352	0.0365	0.019*	
H8BB	−0.0448	0.0819	0.0228	0.019*	
C9B	−0.08761 (12)	−0.08171 (10)	0.12122 (3)	0.01626 (13)	
H9BA	−0.0259	−0.1461	0.1306	0.020*	
H9BB	−0.2050	−0.1375	0.1034	0.020*	
C10B	−0.10482 (11)	−0.00548 (10)	0.16698 (3)	0.01407 (12)	
C11B	−0.23001 (13)	−0.07162 (12)	0.19458 (4)	0.02008 (16)	
H11B	−0.3085	−0.1630	0.1841	0.024*	
C12B	−0.23822 (14)	−0.00165 (13)	0.23783 (4)	0.02261 (18)	
H12B	−0.3215	−0.0450	0.2575	0.027*	
C13B	−0.12295 (13)	0.13227 (12)	0.25180 (3)	0.01974 (16)	
H13B	−0.1257	0.1821	0.2812	0.024*	
C14B	−0.00359 (12)	0.19202 (11)	0.22199 (3)	0.01707 (14)	
H14B	0.0741	0.2843	0.2312	0.020*	
C15B	0.34115 (11)	0.51392 (10)	0.19532 (3)	0.01525 (12)	
C16B	0.39955 (14)	0.64762 (10)	0.22374 (4)	0.01918 (15)	
H16D	0.3243	0.6457	0.2480	0.029*	
H16E	0.3947	0.7185	0.2020	0.029*	
H16F	0.5193	0.6693	0.2404	0.029*	
Cl1	0.33629 (2)	−0.17248 (2)	0.09748 (2)	0.01340 (3)	
O11	0.30474 (11)	−0.12439 (8)	0.14429 (3)	0.01993 (13)	
O12	0.37512 (14)	−0.29685 (10)	0.10216 (4)	0.02729 (17)	
O13	0.47992 (13)	−0.06661 (11)	0.08384 (4)	0.0317 (2)	
O14	0.18341 (12)	−0.19854 (12)	0.06120 (3)	0.0314 (2)	
Cl2	0.66222 (2)	1.15399 (2)	0.40344 (2)	0.01185 (3)	
O21	0.62121 (14)	1.27698 (10)	0.39746 (3)	0.02598 (17)	
O22	0.68767 (11)	1.10012 (8)	0.35669 (2)	0.01847 (12)	
O23	0.81983 (11)	1.18512 (11)	0.43829 (3)	0.02691 (17)	
O24	0.52286 (13)	1.05033 (11)	0.42000 (4)	0.0321 (2)	
Cl3	0.21799 (3)	0.50962 (2)	−0.16092 (2)	0.01582 (3)	
O31	0.27818 (11)	0.65914 (9)	−0.16224 (4)	0.02509 (16)	
O32	0.26294 (13)	0.44580 (11)	−0.20132 (4)	0.0314 (2)	
O33	0.03501 (14)	0.46092 (13)	−0.16261 (6)	0.0477 (3)	
O34	0.3044 (2)	0.48292 (17)	−0.11636 (4)	0.0603 (4)	
Cl4	1.18034 (5)	0.48638 (4)	0.33410 (2)	0.01448 (5)	0.9090 (15)
O41	1.28183 (13)	0.63107 (11)	0.35110 (4)	0.0286 (2)	0.9090 (15)
O42	1.06676 (17)	0.43235 (13)	0.36849 (4)	0.0339 (3)	0.9090 (15)
O43	1.07584 (18)	0.47935 (13)	0.28758 (4)	0.0358 (3)	0.9090 (15)
O44	1.29117 (16)	0.40816 (15)	0.32951 (7)	0.0499 (4)	0.9090 (15)
Cl4A	1.2271 (6)	0.4988 (5)	0.33959 (14)	0.0173 (6)	0.0910 (15)
O41A	1.3201 (11)	0.4703 (10)	0.3811 (3)	0.0286 (2)	0.0910 (15)
O42A	1.0388 (13)	0.4473 (12)	0.3392 (4)	0.0339 (3)	0.0910 (15)
O43A	1.280 (2)	0.6441 (11)	0.3365 (4)	0.0358 (3)	0.0910 (15)
O44A	1.2617 (16)	0.4390 (15)	0.2955 (5)	0.0499 (4)	0.0910 (15)

### Atomic displacement parameters (Å^2^)

**Table:**

	*U*^11^	*U*^22^	*U*^33^	*U*^12^	*U*^13^	*U*^23^
Zn1	0.00995 (3)	0.01079 (4)	0.00945 (3)	0.00291 (3)	0.00217 (2)	0.00021 (2)
O1W	0.0134 (2)	0.0150 (3)	0.0161 (2)	0.00531 (19)	0.00066 (17)	0.00013 (19)
N1A	0.0103 (2)	0.0137 (3)	0.0118 (2)	0.00480 (18)	0.00263 (16)	0.00136 (18)
N2A	0.0126 (2)	0.0133 (3)	0.0137 (2)	0.00588 (19)	0.00452 (18)	0.00143 (19)
N3A	0.0122 (2)	0.0120 (3)	0.0117 (2)	0.00456 (18)	0.00275 (17)	0.00024 (18)
N4A	0.0133 (2)	0.0135 (3)	0.0111 (2)	0.00355 (19)	0.00376 (17)	0.00090 (19)
N5A	0.0180 (3)	0.0136 (3)	0.0177 (3)	0.0034 (2)	0.0025 (2)	−0.0018 (2)
C1A	0.0117 (2)	0.0181 (3)	0.0132 (2)	0.0066 (2)	0.0026 (2)	0.0009 (2)
C2A	0.0131 (3)	0.0183 (4)	0.0145 (3)	0.0051 (2)	0.0049 (2)	0.0000 (2)
C3A	0.0158 (3)	0.0153 (3)	0.0131 (2)	0.0029 (2)	0.0051 (2)	0.0007 (2)
C4A	0.0148 (3)	0.0144 (3)	0.0135 (2)	0.0046 (2)	0.0035 (2)	0.0033 (2)
C5A	0.0113 (2)	0.0116 (3)	0.0127 (2)	0.0041 (2)	0.00288 (18)	0.0020 (2)
C6A	0.0145 (3)	0.0142 (3)	0.0171 (3)	0.0076 (2)	0.0049 (2)	0.0045 (2)
C7A	0.0099 (2)	0.0168 (3)	0.0187 (3)	0.0051 (2)	0.0023 (2)	0.0030 (3)
C8A	0.0135 (3)	0.0137 (3)	0.0139 (2)	0.0036 (2)	−0.0004 (2)	−0.0005 (2)
C9A	0.0178 (3)	0.0105 (3)	0.0159 (3)	0.0035 (2)	0.0057 (2)	0.0013 (2)
C10A	0.0123 (2)	0.0136 (3)	0.0128 (2)	0.0036 (2)	0.00367 (19)	0.0027 (2)
C11A	0.0172 (3)	0.0194 (4)	0.0182 (3)	0.0031 (3)	0.0079 (3)	0.0049 (3)
C12A	0.0199 (4)	0.0275 (5)	0.0178 (3)	0.0067 (3)	0.0104 (3)	0.0061 (3)
C13A	0.0210 (3)	0.0264 (5)	0.0127 (3)	0.0106 (3)	0.0071 (2)	0.0024 (3)
C14A	0.0175 (3)	0.0184 (4)	0.0118 (2)	0.0056 (3)	0.0044 (2)	−0.0004 (2)
C15A	0.0168 (3)	0.0122 (3)	0.0139 (3)	0.0035 (2)	0.0034 (2)	0.0007 (2)
C16A	0.0287 (4)	0.0117 (3)	0.0144 (3)	0.0040 (3)	0.0043 (3)	−0.0005 (2)
Zn2	0.01021 (3)	0.01191 (4)	0.00994 (3)	0.00248 (3)	0.00271 (2)	−0.00051 (3)
O2W	0.0141 (2)	0.0145 (3)	0.0153 (2)	0.00460 (19)	0.00156 (17)	0.00032 (19)
N1B	0.0109 (2)	0.0138 (3)	0.0114 (2)	0.00427 (18)	0.00268 (16)	0.00160 (18)
N2B	0.0135 (2)	0.0148 (3)	0.0167 (2)	0.0067 (2)	0.00503 (19)	0.0004 (2)
N3B	0.0122 (2)	0.0128 (3)	0.0132 (2)	0.00396 (19)	0.00343 (17)	−0.00034 (19)
N4B	0.0135 (2)	0.0156 (3)	0.0123 (2)	0.0037 (2)	0.00466 (18)	0.0001 (2)
N5B	0.0182 (3)	0.0147 (3)	0.0184 (3)	0.0034 (2)	0.0023 (2)	−0.0026 (2)
C1B	0.0124 (2)	0.0177 (3)	0.0118 (2)	0.0061 (2)	0.00272 (19)	0.0019 (2)
C2B	0.0137 (3)	0.0192 (4)	0.0123 (2)	0.0053 (2)	0.0041 (2)	0.0012 (2)
C3B	0.0167 (3)	0.0167 (4)	0.0117 (2)	0.0033 (2)	0.0031 (2)	0.0022 (2)
C4B	0.0162 (3)	0.0156 (3)	0.0150 (3)	0.0048 (2)	0.0023 (2)	0.0046 (2)
C5B	0.0119 (2)	0.0125 (3)	0.0143 (2)	0.0041 (2)	0.0022 (2)	0.0023 (2)
C6B	0.0163 (3)	0.0150 (3)	0.0219 (3)	0.0084 (3)	0.0058 (3)	0.0050 (3)
C7B	0.0100 (2)	0.0192 (4)	0.0218 (3)	0.0054 (2)	0.0023 (2)	0.0023 (3)
C8B	0.0141 (3)	0.0157 (3)	0.0154 (3)	0.0027 (2)	−0.0006 (2)	−0.0012 (2)
C9B	0.0174 (3)	0.0122 (3)	0.0182 (3)	0.0027 (2)	0.0063 (2)	0.0006 (2)
C10B	0.0128 (3)	0.0155 (3)	0.0144 (3)	0.0045 (2)	0.0045 (2)	0.0030 (2)
C11B	0.0177 (3)	0.0209 (4)	0.0202 (3)	0.0021 (3)	0.0088 (3)	0.0057 (3)
C12B	0.0205 (4)	0.0302 (5)	0.0194 (3)	0.0078 (3)	0.0113 (3)	0.0085 (3)
C13B	0.0209 (4)	0.0276 (5)	0.0140 (3)	0.0105 (3)	0.0080 (3)	0.0038 (3)
C14B	0.0184 (3)	0.0202 (4)	0.0128 (3)	0.0059 (3)	0.0059 (2)	−0.0001 (2)
C15B	0.0161 (3)	0.0132 (3)	0.0156 (3)	0.0039 (2)	0.0032 (2)	0.0000 (2)
C16B	0.0255 (4)	0.0125 (3)	0.0177 (3)	0.0040 (3)	0.0054 (3)	−0.0012 (3)
Cl1	0.01268 (6)	0.01273 (7)	0.01505 (6)	0.00419 (5)	0.00425 (5)	0.00015 (5)
O11	0.0276 (3)	0.0176 (3)	0.0170 (2)	0.0092 (3)	0.0090 (2)	−0.0001 (2)
O12	0.0400 (5)	0.0205 (4)	0.0306 (4)	0.0195 (4)	0.0129 (3)	0.0039 (3)
O13	0.0281 (4)	0.0244 (4)	0.0381 (5)	−0.0021 (3)	0.0190 (4)	0.0039 (4)
O14	0.0241 (4)	0.0441 (6)	0.0252 (4)	0.0174 (4)	−0.0069 (3)	−0.0100 (4)
Cl2	0.01223 (6)	0.01228 (7)	0.01196 (6)	0.00475 (5)	0.00369 (5)	0.00156 (5)
O21	0.0431 (5)	0.0244 (4)	0.0232 (3)	0.0248 (4)	0.0125 (3)	0.0065 (3)
O22	0.0265 (3)	0.0180 (3)	0.0133 (2)	0.0101 (2)	0.0060 (2)	−0.0003 (2)
O23	0.0217 (3)	0.0375 (5)	0.0204 (3)	0.0139 (3)	−0.0057 (2)	−0.0068 (3)
O24	0.0290 (4)	0.0255 (4)	0.0368 (5)	−0.0031 (3)	0.0195 (4)	0.0047 (4)
Cl3	0.01840 (8)	0.01536 (8)	0.01327 (6)	0.00440 (6)	0.00522 (5)	0.00059 (6)
O31	0.0211 (3)	0.0159 (3)	0.0381 (4)	0.0051 (2)	0.0087 (3)	0.0000 (3)
O32	0.0294 (4)	0.0306 (5)	0.0326 (4)	0.0080 (3)	0.0116 (3)	−0.0113 (4)
O33	0.0229 (4)	0.0339 (6)	0.0813 (10)	−0.0011 (4)	0.0264 (5)	−0.0116 (6)
O34	0.0904 (11)	0.0514 (8)	0.0264 (5)	0.0168 (8)	−0.0155 (6)	0.0158 (5)
Cl4	0.01332 (12)	0.01540 (11)	0.01515 (10)	0.00505 (11)	0.00474 (10)	−0.00139 (8)
O41	0.0188 (3)	0.0219 (4)	0.0380 (5)	−0.0011 (3)	0.0079 (4)	−0.0122 (4)
O42	0.0441 (6)	0.0305 (5)	0.0285 (4)	0.0074 (4)	0.0227 (4)	0.0075 (4)
O43	0.0524 (7)	0.0308 (6)	0.0174 (3)	0.0114 (5)	−0.0060 (4)	−0.0023 (3)
O44	0.0274 (5)	0.0380 (7)	0.0897 (12)	0.0219 (5)	0.0089 (6)	−0.0136 (7)
Cl4A	0.0147 (13)	0.0239 (15)	0.0141 (10)	0.0072 (13)	0.0038 (10)	0.0015 (9)
O41A	0.0188 (3)	0.0219 (4)	0.0380 (5)	−0.0011 (3)	0.0079 (4)	−0.0122 (4)
O42A	0.0441 (6)	0.0305 (5)	0.0285 (4)	0.0074 (4)	0.0227 (4)	0.0075 (4)
O43A	0.0524 (7)	0.0308 (6)	0.0174 (3)	0.0114 (5)	−0.0060 (4)	−0.0023 (3)
O44A	0.0274 (5)	0.0380 (7)	0.0897 (12)	0.0219 (5)	0.0089 (6)	−0.0136 (7)

### Geometric parameters (Å, º)

**Table:**

Zn1—N5A	2.1231 (8)	N2B—C6B	1.4730 (12)
Zn1—O1W	2.1333 (7)	N2B—C7B	1.4737 (12)
Zn1—N4A	2.1341 (7)	N2B—H2BB	1.0000
Zn1—N2A	2.1692 (7)	N3B—C9B	1.4734 (11)
Zn1—N1A	2.1707 (7)	N3B—C8B	1.4818 (12)
Zn1—N3A	2.2056 (7)	N3B—H3BB	1.0000
O1W—H1W1	0.839 (13)	N4B—C10B	1.3413 (12)
O1W—H1W2	0.803 (13)	N4B—C14B	1.3458 (11)
N1A—C5A	1.3441 (11)	N5B—C15B	1.1464 (12)
N1A—C1A	1.3443 (10)	C1B—C2B	1.3861 (11)
N2A—C6A	1.4711 (11)	C1B—H1BA	0.9500
N2A—C7A	1.4757 (12)	C2B—C3B	1.3915 (13)
N2A—H2AB	1.0000	C2B—H2BA	0.9500
N3A—C9A	1.4754 (11)	C3B—C4B	1.3861 (13)
N3A—C8A	1.4811 (11)	C3B—H3BA	0.9500
N3A—H3AB	1.0000	C4B—C5B	1.3945 (12)
N4A—C10A	1.3415 (11)	C4B—H4BA	0.9500
N4A—C14A	1.3476 (11)	C5B—C6B	1.5111 (12)
N5A—C15A	1.1457 (12)	C6B—H6BA	0.9900
C1A—C2A	1.3892 (11)	C6B—H6BB	0.9900
C1A—H1AA	0.9500	C7B—C8B	1.5222 (14)
C2A—C3A	1.3917 (14)	C7B—H7BA	0.9900
C2A—H2AA	0.9500	C7B—H7BB	0.9900
C3A—C4A	1.3867 (13)	C8B—H8BA	0.9900
C3A—H3AA	0.9500	C8B—H8BB	0.9900
C4A—C5A	1.3954 (11)	C9B—C10B	1.5099 (12)
C4A—H4AA	0.9500	C9B—H9BA	0.9900
C5A—C6A	1.5094 (11)	C9B—H9BB	0.9900
C6A—H6AA	0.9900	C10B—C11B	1.3925 (12)
C6A—H6AB	0.9900	C11B—C12B	1.3917 (15)
C7A—C8A	1.5204 (13)	C11B—H11B	0.9500
C7A—H7AA	0.9900	C12B—C13B	1.3873 (17)
C7A—H7AB	0.9900	C12B—H12B	0.9500
C8A—H8AA	0.9900	C13B—C14B	1.3874 (13)
C8A—H8AB	0.9900	C13B—H13B	0.9500
C9A—C10A	1.5067 (12)	C14B—H14B	0.9500
C9A—H9AA	0.9900	C15B—C16B	1.4456 (13)
C9A—H9AB	0.9900	C16B—H16D	0.9800
C10A—C11A	1.3933 (12)	C16B—H16E	0.9800
C11A—C12A	1.3891 (14)	C16B—H16F	0.9800
C11A—H11A	0.9500	Cl1—O12	1.4317 (9)
C12A—C13A	1.3925 (16)	Cl1—O13	1.4334 (9)
C12A—H12A	0.9500	Cl1—O14	1.4399 (9)
C13A—C14A	1.3880 (12)	Cl1—O11	1.4577 (7)
C13A—H13A	0.9500	Cl2—O21	1.4313 (9)
C14A—H14A	0.9500	Cl2—O24	1.4371 (9)
C15A—C16A	1.4499 (13)	Cl2—O23	1.4399 (8)
C16A—H16A	0.9800	Cl2—O22	1.4574 (7)
C16A—H16B	0.9800	Cl3—O33	1.4227 (10)
C16A—H16C	0.9800	Cl3—O34	1.4272 (11)
Zn2—O2W	2.1279 (7)	Cl3—O32	1.4289 (9)
Zn2—N5B	2.1328 (8)	Cl3—O31	1.4575 (9)
Zn2—N4B	2.1345 (7)	Cl4—O44	1.4263 (12)
Zn2—N1B	2.1440 (7)	Cl4—O43	1.4334 (11)
Zn2—N2B	2.1674 (8)	Cl4—O42	1.4396 (10)
Zn2—N3B	2.2066 (7)	Cl4—O41	1.4592 (11)
O2W—H2W1	0.818 (13)	Cl4A—O41A	1.394 (10)
O2W—H2W2	0.817 (13)	Cl4A—O43A	1.425 (11)
N1B—C1B	1.3436 (11)	Cl4A—O44A	1.451 (11)
N1B—C5B	1.3451 (11)	Cl4A—O42A	1.470 (10)
			
N5A—Zn1—O1W	86.48 (3)	Zn2—O2W—H2W1	115.0 (14)
N5A—Zn1—N4A	93.75 (3)	Zn2—O2W—H2W2	120.0 (12)
O1W—Zn1—N4A	95.55 (3)	H2W1—O2W—H2W2	106.0 (16)
N5A—Zn1—N2A	92.19 (3)	C1B—N1B—C5B	118.86 (7)
O1W—Zn1—N2A	172.06 (3)	C1B—N1B—Zn2	126.05 (6)
N4A—Zn1—N2A	92.34 (3)	C5B—N1B—Zn2	114.63 (5)
N5A—Zn1—N1A	96.07 (3)	C6B—N2B—C7B	114.68 (7)
O1W—Zn1—N1A	93.28 (3)	C6B—N2B—Zn2	109.28 (5)
N4A—Zn1—N1A	167.17 (3)	C7B—N2B—Zn2	105.72 (6)
N2A—Zn1—N1A	79.06 (3)	C6B—N2B—H2BB	109.0
N5A—Zn1—N3A	171.11 (3)	C7B—N2B—H2BB	109.0
O1W—Zn1—N3A	99.00 (3)	Zn2—N2B—H2BB	109.0
N4A—Zn1—N3A	78.83 (3)	C9B—N3B—C8B	113.46 (7)
N2A—Zn1—N3A	83.32 (3)	C9B—N3B—Zn2	110.38 (5)
N1A—Zn1—N3A	90.62 (3)	C8B—N3B—Zn2	103.99 (6)
Zn1—O1W—H1W1	121.3 (12)	C9B—N3B—H3BB	109.6
Zn1—O1W—H1W2	118.1 (13)	C8B—N3B—H3BB	109.6
H1W1—O1W—H1W2	104.6 (15)	Zn2—N3B—H3BB	109.6
C5A—N1A—C1A	118.48 (7)	C10B—N4B—C14B	118.88 (7)
C5A—N1A—Zn1	113.99 (5)	C10B—N4B—Zn2	115.92 (5)
C1A—N1A—Zn1	126.90 (6)	C14B—N4B—Zn2	124.92 (6)
C6A—N2A—C7A	114.60 (7)	C15B—N5B—Zn2	171.64 (8)
C6A—N2A—Zn1	109.42 (5)	N1B—C1B—C2B	122.58 (8)
C7A—N2A—Zn1	105.92 (5)	N1B—C1B—H1BA	118.7
C6A—N2A—H2AB	108.9	C2B—C1B—H1BA	118.7
C7A—N2A—H2AB	108.9	C1B—C2B—C3B	118.67 (8)
Zn1—N2A—H2AB	108.9	C1B—C2B—H2BA	120.7
C9A—N3A—C8A	113.24 (7)	C3B—C2B—H2BA	120.7
C9A—N3A—Zn1	110.38 (5)	C4B—C3B—C2B	118.97 (8)
C8A—N3A—Zn1	104.29 (5)	C4B—C3B—H3BA	120.5
C9A—N3A—H3AB	109.6	C2B—C3B—H3BA	120.5
C8A—N3A—H3AB	109.6	C3B—C4B—C5B	119.14 (8)
Zn1—N3A—H3AB	109.6	C3B—C4B—H4BA	120.4
C10A—N4A—C14A	118.80 (7)	C5B—C4B—H4BA	120.4
C10A—N4A—Zn1	115.96 (5)	N1B—C5B—C4B	121.76 (8)
C14A—N4A—Zn1	124.98 (6)	N1B—C5B—C6B	116.85 (7)
C15A—N5A—Zn1	173.47 (8)	C4B—C5B—C6B	121.37 (8)
N1A—C1A—C2A	122.56 (8)	N2B—C6B—C5B	112.39 (7)
N1A—C1A—H1AA	118.7	N2B—C6B—H6BA	109.1
C2A—C1A—H1AA	118.7	C5B—C6B—H6BA	109.1
C1A—C2A—C3A	118.96 (8)	N2B—C6B—H6BB	109.1
C1A—C2A—H2AA	120.5	C5B—C6B—H6BB	109.1
C3A—C2A—H2AA	120.5	H6BA—C6B—H6BB	107.9
C4A—C3A—C2A	118.64 (7)	N2B—C7B—C8B	111.43 (7)
C4A—C3A—H3AA	120.7	N2B—C7B—H7BA	109.3
C2A—C3A—H3AA	120.7	C8B—C7B—H7BA	109.3
C3A—C4A—C5A	119.11 (8)	N2B—C7B—H7BB	109.3
C3A—C4A—H4AA	120.4	C8B—C7B—H7BB	109.3
C5A—C4A—H4AA	120.4	H7BA—C7B—H7BB	108.0
N1A—C5A—C4A	122.22 (7)	N3B—C8B—C7B	111.31 (7)
N1A—C5A—C6A	116.98 (7)	N3B—C8B—H8BA	109.4
C4A—C5A—C6A	120.78 (7)	C7B—C8B—H8BA	109.4
N2A—C6A—C5A	112.56 (7)	N3B—C8B—H8BB	109.4
N2A—C6A—H6AA	109.1	C7B—C8B—H8BB	109.4
C5A—C6A—H6AA	109.1	H8BA—C8B—H8BB	108.0
N2A—C6A—H6AB	109.1	N3B—C9B—C10B	112.76 (7)
C5A—C6A—H6AB	109.1	N3B—C9B—H9BA	109.0
H6AA—C6A—H6AB	107.8	C10B—C9B—H9BA	109.0
N2A—C7A—C8A	111.17 (7)	N3B—C9B—H9BB	109.0
N2A—C7A—H7AA	109.4	C10B—C9B—H9BB	109.0
C8A—C7A—H7AA	109.4	H9BA—C9B—H9BB	107.8
N2A—C7A—H7AB	109.4	N4B—C10B—C11B	122.01 (8)
C8A—C7A—H7AB	109.4	N4B—C10B—C9B	118.08 (7)
H7AA—C7A—H7AB	108.0	C11B—C10B—C9B	119.88 (9)
N3A—C8A—C7A	111.08 (7)	C12B—C11B—C10B	118.89 (10)
N3A—C8A—H8AA	109.4	C12B—C11B—H11B	120.6
C7A—C8A—H8AA	109.4	C10B—C11B—H11B	120.6
N3A—C8A—H8AB	109.4	C13B—C12B—C11B	119.03 (8)
C7A—C8A—H8AB	109.4	C13B—C12B—H12B	120.5
H8AA—C8A—H8AB	108.0	C11B—C12B—H12B	120.5
N3A—C9A—C10A	113.01 (7)	C12B—C13B—C14B	118.73 (9)
N3A—C9A—H9AA	109.0	C12B—C13B—H13B	120.6
C10A—C9A—H9AA	109.0	C14B—C13B—H13B	120.6
N3A—C9A—H9AB	109.0	N4B—C14B—C13B	122.44 (9)
C10A—C9A—H9AB	109.0	N4B—C14B—H14B	118.8
H9AA—C9A—H9AB	107.8	C13B—C14B—H14B	118.8
N4A—C10A—C11A	122.03 (8)	N5B—C15B—C16B	178.72 (10)
N4A—C10A—C9A	118.05 (7)	C15B—C16B—H16D	109.5
C11A—C10A—C9A	119.89 (8)	C15B—C16B—H16E	109.5
C12A—C11A—C10A	119.04 (9)	H16D—C16B—H16E	109.5
C12A—C11A—H11A	120.5	C15B—C16B—H16F	109.5
C10A—C11A—H11A	120.5	H16D—C16B—H16F	109.5
C11A—C12A—C13A	119.00 (8)	H16E—C16B—H16F	109.5
C11A—C12A—H12A	120.5	O12—Cl1—O13	110.20 (6)
C13A—C12A—H12A	120.5	O12—Cl1—O14	110.05 (6)
C14A—C13A—C12A	118.56 (8)	O13—Cl1—O14	109.68 (7)
C14A—C13A—H13A	120.7	O12—Cl1—O11	109.31 (5)
C12A—C13A—H13A	120.7	O13—Cl1—O11	108.58 (6)
N4A—C14A—C13A	122.55 (9)	O14—Cl1—O11	108.98 (5)
N4A—C14A—H14A	118.7	O21—Cl2—O24	110.32 (7)
C13A—C14A—H14A	118.7	O21—Cl2—O23	109.89 (6)
N5A—C15A—C16A	178.76 (10)	O24—Cl2—O23	109.62 (7)
C15A—C16A—H16A	109.5	O21—Cl2—O22	109.38 (5)
C15A—C16A—H16B	109.5	O24—Cl2—O22	108.82 (6)
H16A—C16A—H16B	109.5	O23—Cl2—O22	108.78 (5)
C15A—C16A—H16C	109.5	O33—Cl3—O34	110.84 (10)
H16A—C16A—H16C	109.5	O33—Cl3—O32	110.98 (7)
H16B—C16A—H16C	109.5	O34—Cl3—O32	109.27 (9)
O2W—Zn2—N5B	86.23 (3)	O33—Cl3—O31	108.63 (7)
O2W—Zn2—N4B	95.01 (3)	O34—Cl3—O31	107.40 (8)
N5B—Zn2—N4B	94.02 (3)	O32—Cl3—O31	109.64 (6)
O2W—Zn2—N1B	94.46 (3)	O44—Cl4—O43	109.61 (9)
N5B—Zn2—N1B	95.88 (3)	O44—Cl4—O42	110.78 (10)
N4B—Zn2—N1B	166.72 (3)	O43—Cl4—O42	108.45 (8)
O2W—Zn2—N2B	173.16 (3)	O44—Cl4—O41	110.85 (8)
N5B—Zn2—N2B	91.53 (3)	O43—Cl4—O41	108.89 (8)
N4B—Zn2—N2B	91.59 (3)	O42—Cl4—O41	108.19 (7)
N1B—Zn2—N2B	79.32 (3)	O41A—Cl4A—O43A	110.6 (7)
O2W—Zn2—N3B	99.39 (3)	O41A—Cl4A—O44A	110.7 (7)
N5B—Zn2—N3B	171.13 (3)	O43A—Cl4A—O44A	106.2 (8)
N4B—Zn2—N3B	78.74 (3)	O41A—Cl4A—O42A	112.6 (6)
N1B—Zn2—N3B	90.55 (3)	O43A—Cl4A—O42A	107.6 (8)
N2B—Zn2—N3B	83.64 (3)	O44A—Cl4A—O42A	108.9 (7)
			
C5A—N1A—C1A—C2A	−0.45 (13)	C5B—N1B—C1B—C2B	0.17 (13)
Zn1—N1A—C1A—C2A	−170.73 (7)	Zn2—N1B—C1B—C2B	171.97 (7)
N1A—C1A—C2A—C3A	1.40 (14)	N1B—C1B—C2B—C3B	−0.97 (14)
C1A—C2A—C3A—C4A	−0.62 (14)	C1B—C2B—C3B—C4B	0.37 (14)
C2A—C3A—C4A—C5A	−0.99 (13)	C2B—C3B—C4B—C5B	0.95 (14)
C1A—N1A—C5A—C4A	−1.28 (13)	C1B—N1B—C5B—C4B	1.23 (13)
Zn1—N1A—C5A—C4A	170.23 (7)	Zn2—N1B—C5B—C4B	−171.48 (7)
C1A—N1A—C5A—C6A	176.98 (8)	C1B—N1B—C5B—C6B	−177.17 (8)
Zn1—N1A—C5A—C6A	−11.51 (10)	Zn2—N1B—C5B—C6B	10.12 (10)
C3A—C4A—C5A—N1A	2.01 (13)	C3B—C4B—C5B—N1B	−1.80 (14)
C3A—C4A—C5A—C6A	−176.18 (8)	C3B—C4B—C5B—C6B	176.53 (9)
C7A—N2A—C6A—C5A	88.34 (9)	C7B—N2B—C6B—C5B	−88.80 (9)
Zn1—N2A—C6A—C5A	−30.44 (8)	Zn2—N2B—C6B—C5B	29.66 (9)
N1A—C5A—C6A—N2A	28.85 (11)	N1B—C5B—C6B—N2B	−27.36 (11)
C4A—C5A—C6A—N2A	−152.86 (8)	C4B—C5B—C6B—N2B	154.22 (8)
C6A—N2A—C7A—C8A	−81.07 (9)	C6B—N2B—C7B—C8B	80.96 (10)
Zn1—N2A—C7A—C8A	39.66 (8)	Zn2—N2B—C7B—C8B	−39.49 (8)
C9A—N3A—C8A—C7A	−78.77 (9)	C9B—N3B—C8B—C7B	79.08 (9)
Zn1—N3A—C8A—C7A	41.26 (7)	Zn2—N3B—C8B—C7B	−40.87 (8)
N2A—C7A—C8A—N3A	−57.67 (9)	N2B—C7B—C8B—N3B	57.50 (10)
C8A—N3A—C9A—C10A	95.69 (9)	C8B—N3B—C9B—C10B	−94.37 (9)
Zn1—N3A—C9A—C10A	−20.81 (9)	Zn2—N3B—C9B—C10B	21.88 (9)
C14A—N4A—C10A—C11A	−0.70 (13)	C14B—N4B—C10B—C11B	0.15 (14)
Zn1—N4A—C10A—C11A	173.79 (7)	Zn2—N4B—C10B—C11B	−174.13 (8)
C14A—N4A—C10A—C9A	177.04 (8)	C14B—N4B—C10B—C9B	−177.88 (9)
Zn1—N4A—C10A—C9A	−8.47 (10)	Zn2—N4B—C10B—C9B	7.84 (11)
N3A—C9A—C10A—N4A	20.16 (11)	N3B—C9B—C10B—N4B	−20.49 (12)
N3A—C9A—C10A—C11A	−162.05 (8)	N3B—C9B—C10B—C11B	161.43 (9)
N4A—C10A—C11A—C12A	1.57 (15)	N4B—C10B—C11B—C12B	−0.97 (16)
C9A—C10A—C11A—C12A	−176.13 (9)	C9B—C10B—C11B—C12B	177.03 (10)
C10A—C11A—C12A—C13A	−0.78 (16)	C10B—C11B—C12B—C13B	0.76 (17)
C11A—C12A—C13A—C14A	−0.78 (16)	C11B—C12B—C13B—C14B	0.20 (17)
C10A—N4A—C14A—C13A	−0.98 (14)	C10B—N4B—C14B—C13B	0.88 (15)
Zn1—N4A—C14A—C13A	−174.93 (7)	Zn2—N4B—C14B—C13B	174.61 (8)
C12A—C13A—C14A—N4A	1.72 (15)	C12B—C13B—C14B—N4B	−1.06 (16)

### Hydrogen-bond geometry (Å, º)

**Table:**

*D*—H···*A*	*D*—H	H···*A*	*D*···*A*	*D*—H···*A*
O1*W*—H1*W*2···O41^i^	0.80 (1)	2.04 (1)	2.8410 (12)	178 (2)
O1*W*—H1*W*2···O43*A*^i^	0.80 (1)	1.94 (2)	2.730 (15)	170 (2)
N2*A*—H2*AB*···O41	1.00	2.28	3.1809 (12)	150
N2*A*—H2*AB*···O42	1.00	2.28	3.1468 (15)	144
N2*A*—H2*AB*···O42*A*	1.00	2.40	3.328 (12)	153
N2*A*—H2*AB*···O43*A*	1.00	2.50	3.396 (15)	149
N3*A*—H3*AB*···O23	1.00	2.17	3.0889 (12)	152
C4*A*—H4*AA*···O42^ii^	0.95	2.60	3.4025 (15)	142
C7*A*—H7*AA*···O24^iii^	0.99	2.41	3.1218 (12)	128
C14*A*—H14*A*···O32^iv^	0.95	2.41	3.1645 (13)	136
C16*A*—H16*B*···O22^v^	0.98	2.51	3.4788 (13)	169
O2*W*—H2*W*1···O31^iv^	0.82 (1)	2.00 (1)	2.8091 (11)	174 (2)
O2*W*—H2*W*2···O11	0.82 (1)	1.99 (1)	2.7690 (11)	159 (2)
N2*B*—H2*BB*···O31^vi^	1.00	2.36	3.2467 (12)	148
N2*B*—H2*BB*···O33^vi^	1.00	2.32	3.2331 (15)	152
N3*B*—H3*BB*···O14	1.00	2.20	3.1169 (13)	152
C7*B*—H7*BB*···O13^i^	0.99	2.44	3.1430 (12)	128
C14*B*—H14*B*···O43^i^	0.95	2.48	3.2138 (15)	134
C14*B*—H14*B*···O44*A*^i^	0.95	2.32	3.149 (13)	145
C16*B*—H16*D*···O43*A*^i^	0.98	2.54	3.419 (13)	150
C16*B*—H16*E*···O11^vii^	0.98	2.55	3.5083 (14)	166

Symmetry codes: (i) *x*−1, *y*, *z*; (ii) −*x*+2, −*y*+1, −*z*+1; (iii) *x*+1, *y*, *z*; (iv) −*x*+1, −*y*+1, −*z*; (v) *x*, *y*−1, *z*; (vi) −*x*, −*y*+1, −*z*; (vii) *x*, *y*+1, *z*.
